# Association between triglyceride-glucose index and its composite obesity indexes and cardio-renal disease: analysis of the NHANES 2013-2018 cycle

**DOI:** 10.3389/fendo.2025.1505808

**Published:** 2025-01-31

**Authors:** Yu Wu, Chengsen Liu, Jiandong Cao

**Affiliations:** ^1^ Department of Oncology, Huainan Xinhua Medical Group Xinhua Hospital, Huainan, Anhui, China; ^2^ Department of Radiotherapy, The People’s Hospital of Liaoning Province, Shenyang, Liaoning, China; ^3^ Department of Thoracic Surgery, Shenyang Chest Hospital & Tenth People’s Hospital, Shenyang, Liaoning, China

**Keywords:** TyG related indicators, chronic kidney disease, cardiovascular disease, cardiorenal syndrome, NHANES

## Abstract

**Background:**

The association between triglyceride-glucose (TYG) and its composite obesity indexes and cardio-renal disease in the American population remains insufficiently researched.

**Methods:**

This study examined a cohort of 11,491 American adults aged 20 years and older from the 2003-2018 National Health and Nutrition Examination Survey (NHANES). To explore the associations between TYG, TyG-Waist-to-Height Ratio (TyG-WHtR), TyG-Body Mass Index (TyG-BMI), TyG-Waist Circumference (TyG-WC), and chronic kidney disease (CKD), cardiovascular disease (CVD), and cardiorenal syndrome (CRS), we utilized weighted multivariate logistic regression, restricted cubic spline (RCS), Receiver operating characteristic (ROC), and subgroup analyses.

**Results:**

Adjusted for confounding factors, there are positive associations between the likelihood of CKD, CVD, and CRS, as well as TYG and its composite obesity indexes. The TYG index was correlated most strongly with CKD (OR 1.42, 95% CI 1.11, 1.82; P = 0.007), while TyG-WHtR had the strongest correlations with CVD (OR 1.63, 95% CI 1.19, 2.22; P = 0.003) and CRS (OR 1.44, 95% CI 1.00, 2.08; P = 0.055). A nonlinear connection was found by RCS analysis between TYG and its composite obesity indexes with CKD (P for overall < 0.001, P for nonlinear < 0.05), while the association with CVD and CRS was predominantly linear (P for overall < 0.001, P for nonlinear > 0.05). Based on ROC curves, TyG-WHtR and TyG-WC emerged as more reliable diagnostic tools than TYG for cardiac and renal diseases. According to subgroup analyses, TyG and its composite obesity measurements were more strongly associated with CKD in younger individuals (≤ 50), males, and those with diabetes mellitus (P for interaction 0.05).

**Conclusions:**

The TyG-WHtR and TyG-WC are associated with an increased risk of cardiac and renal disease, indicating enhanced diagnostic accuracy. These metrics provide an effective tool for identifying early cardiorenal disease and improving risk stratification.

## Introduction

Chronic Kidney Disease (CKD) and Cardiovascular Disease (CVD) are substantial factors contributing to the loss of healthy life expectancy and the high burden on healthcare systems. These are global public health challenges (1). There is a tight association between CKD and CVD, with CKD patients being at high risk for CVD, and CKD being a major cause of death among these patients (2). Cardiorenal syndrome (CRS) is a clinical condition characterized by complex interactions between the heart and kidneys. According to reports, Compared to the general population, CRS sufferers have a 10-30 times higher death rate (3, 4). Therefore, it is increasingly necessary for research to investigate the potential influencing variables and screening indicators of cardiorenal disease. This research may help in the timely detection and diagnosis of cardiorenal illness, ultimately lowering the risk of its occurrences.

Insulin resistance (IR) is a state in which the body is insensitive to the action of insulin, resulting in a decrease in insulin’s efficacy in promoting glucose uptake and utilization. As a result, the body adjusts by developing metabolic abnormalities such as hyperinsulinemia (5). IR is involved in the development of numerous metabolic illnesses (6), including cardiovascular disease (7), obesity (8), and metabolic syndrome (9). The Triglyceride-Glucose (TyG) index evaluates the body’s insulin sensitivity by computing the logarithmic product of TG and fasting glucose. It is regarded as a new and trustworthy predictor of IR (10, 11). Previous studies have shown a significant association between TyG index and chronic kidney disease and adverse cardiovascular events. For example, a cohort study from China found a nonlinear correlation between TyG index and CKD, suggesting that excessively high or low TyG index may lead to an increased risk of developing CDK (12). Similarly, a higher baseline TyG index was identified as a predictor of severity in coronary patients (13). Similar findings have been observed in other ethnically diverse countries. This validates the clinical utility of the index (14, 15). Obesity is widespread globally and is closely associated with a number of health hazards, leading to the onset and progression of chronic diseases such as heart and kidney disease (16, 17). Several studies indicate that the TyG Joint Obesity indexes perform better than the TyG index (18, 19). Therefore, it is crucial to further explore the association between TyG and its associated various obesity indicators and cardio-renal disease.

The kidney and heart are crucial target organs for metabolic illnesses. However, previous studies have focused on cardiovascular disease in Asian and European populations. Studies on the correlation between TyG and cardiorenal syndrome in US populations are scarce (15, 20). In addition, there are few studies on the differences between various TyG-related indicators of obesity in predicting the risk of cardiorenal disease. This study utilized the National Health and Nutrition Examination Survey (NHANES) database to examine the relationship between the TyG index, its composite obesity indexes, and CKD, CVD, and CRS. To investigate the differences in these indicators in predicting the risk of cardiorenal disease risk, with a view to providing a scientific basis for the early prevention and identification of cardiorenal disease.

## Materials and methods

### Study design and participants

**Figure 1:**
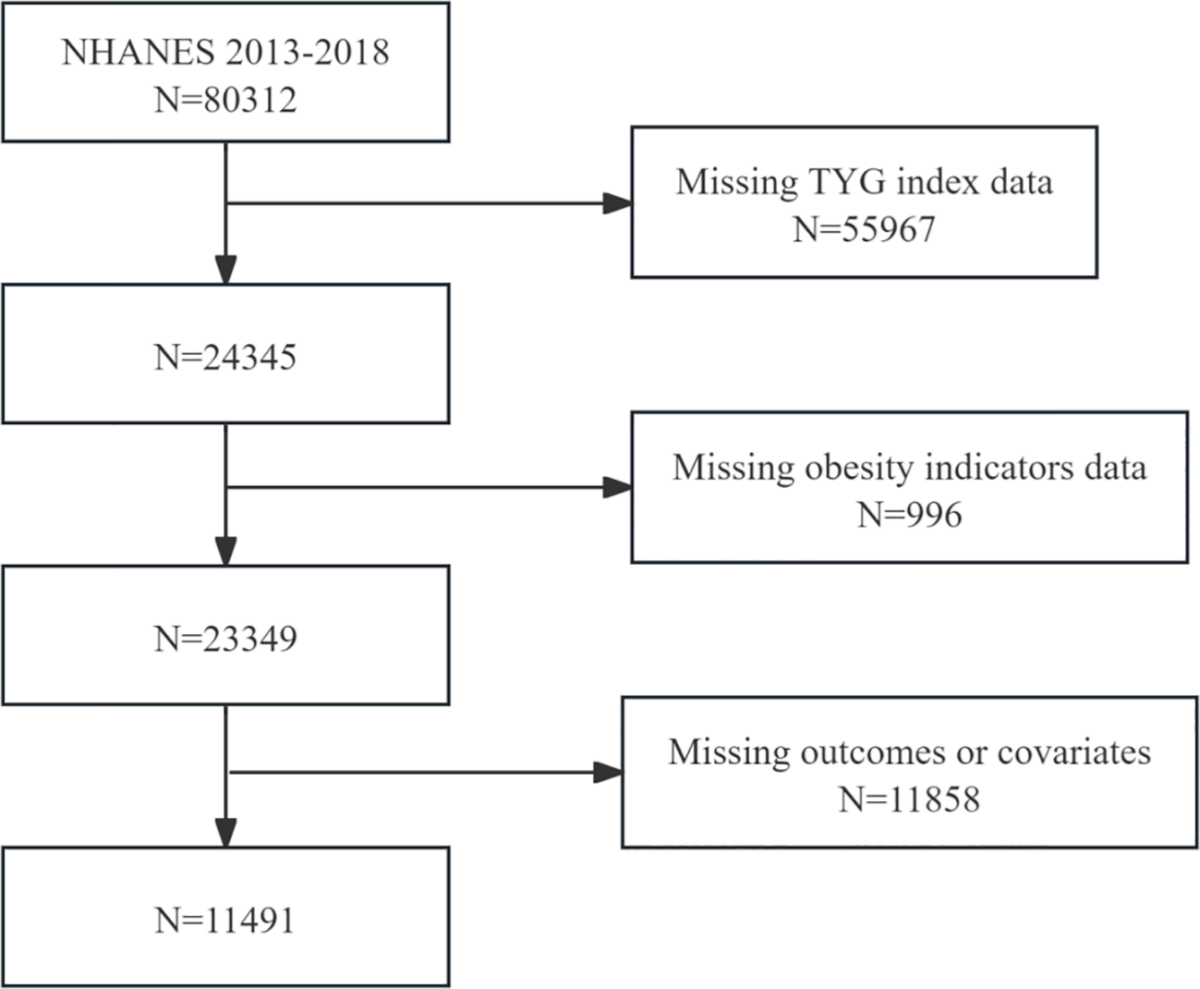
Flowchart depicting participant selection.

This study utilized NHANES data from eight consecutive survey rounds from 2003-2018, comprising a total of 80,312 initial samples. For populations with missing data, since the data were missing at random and the sample size was relatively large, we followed the methodology of the previous study and excluded them from subsequent analyses: (1) missing TyG index (N=55,967); (2) missing related obesity indicators (N=996); and (3) missing endpoints or covariates (N=11,858).The final 11,491 eligible adult participants were enrolled in the study. **Figure 1** depicts the comprehensive selection procedure. Detailed information about the NHANES study procedures was obtained from the National Center for Health Statistics Research Ethics Review Board. Participants provided written consent before participation in the study was initiated.

### Calculation of TyG, TyG-WC, TyG-WHtR, and TyG-BMI

Four metrics were constructed in the study: (1) TyG = ln [triglycerides (mg/dL) × glucose (mg/dL)/2]; (2) TyG-WC = TyG × waist circumference; (3) TyG-WHtR = TyG × waist circumference/height; (4) TyG-BMI = TyG × BMI (21).

### CVD, CVD, and CRS ascertainment

Referring to prior studies on NHANES participants, CKD is indicated if the urine albumin-creatinine ratio (UACR) is ≥30 mg/g and/or the glomerular filtration rate (GFR) is <60 mL/min/1.73 m². Calculation of GFR based on serum creatinine was performed using the CKD-EPI equation. To determine the random UACR, the urine albumin concentration is divided by the urine creatinine concentration (22). CVD is defined according to the NHANES MCQ questionnaire, in which participants answered “yes” to any of the questions in variable names 160b-f (23). Individuals with both CVD and CKD are classified CRS (24).

### Assessment of covariates

The variable age was considered continuous, while gender was categorized into two groups: male and female. Race and ethnicity were grouped using RIDRETH1, and individuals were classified based on marital status into groups of married, those living with a partner, and single individuals. The poverty income ratio (PIR) was divided into groups of <1, 1-3, and ≥3, with smoking status grouped according to whether individuals had smoked more than 100 cigarettes in their lifetime, and drinking status grouped according to whether they consumed fewer than 12 drinks per year. Using a standardized questionnaire data, self-reported physician diagnoses of cancer, hypertension, and diabetes mellitus were ascertained. Physical examinations were conducted to measure weight, height, blood pressure, waist circumference, and BMI. Laboratory blood tests were used to measure total cholesterol, TG, and fasting blood glucose (FBG), all of which were regarded as continuous variables.

### Statistical analysis

Statistics were calculated in accordance with the CDC’s guidelines. Sample weights, clustering, and stratification were all included in the analyses for this study, as NHANES uses a complex probabilistic sampling methodology. The weighted means (95% CI) of continuous variables are presented, while categorical variables are presented in weighted percentage format (95% CI). The association between different TyG-related variables and CKD, CVD, and CRS was evaluated using multivariate logistic regression. Three adjustment models were developed to ensure a comprehensive analysis: Model 1 has no covariates adjusted for; Model 2 adjusts for demographic variables of age, sex, and race; and Model 3 further adjusts for marital status, PIR, alcohol consumption, smoking, cancer, hypertension, diabetes, and total cholesterol. To investigate the nonlinear dose-response relationship between TyG, related obesity, and CKD, CVD, and CRS, restricted cubic spline (RCS) curve analyses were performed at the 5th, 35th, 65th, and 95th percentiles of the distribution of TyG and related obesity, adjusting for the same variables as in Model 3, with 4 nodes set to reduce the potential impact of outliers. Additionally, in Model 3, diagnostic value analysis was performed using subject ROC curves. Finally, subgroup analyses based on fully adjusted models were conducted to examine associations between TyG and its composite obesity indexes with CKD, CVD, and CRS across different subgroups of age, sex, race, alcohol consumption, smoking, hypertension, and diabetes. Each of the statistical evaluations was performed using R (version 4.1.3) and EmpowerStats (version 6.0). Statistical significance was set at P < 0.05.

## Results

### Basic characteristics

The baseline characteristics of individuals selected from NHANES between 2003 and 2018 are compiled in **Table 1** and the supplementary materials, **Supplementary Tables S1–S3**. A total of 11,491 participants were enrolled, and individuals were categorized according to the quartiles of TyG and its composite obesity indexes. Higher TyG and its composite obesity indexes were linked to older age, a higher percentage of males, increased BMI, WC, TG, fasting glucose levels, alcohol and tobacco consumption, as well as the presence of diabetes mellitus, hypertension, cancer, CKD, CVD, and CRS among participants.

**Table 1:** Baseline characteristics of the individuals selected from NHANES between 2003 and 2018.

	Triglyceride-glucose (TyG) (N = 11,491)				
	Q1 (5.65–8.17)	Q2 (8.17–8.59)	Q3 (8.59–9.04)	Q4 (9.04–12.85)	P-value
Age, years	42.38 (41.38,43.38)	47.38 (46.55,48.21)	49.43 (48.68,50.18)	51.94 (51.19,52.69)	<0.0001
Height, cm	168.41 (167.89,168.93)	169.42 (168.96,169.88)	169.07 (168.58,169.56)	169.79 (169.32,170.25)	0.0009
BMI, kg/m2	26.34 (25.99,26.68)	28.38 (28.09,28.68)	30.20 (29.82,30.57)	31.82 (31.46,32.19)	<0.0001
Waist circumference, cm	91.03 (90.18,91.87)	97.74 (96.92,98.56)	102.89 (102.02,103.76)	108.04 (107.19,108.88)	<0.0001
Fasting glucose, mmol/L	5.25 (5.21,5.28)	5.52 (5.49,5.56)	5.79 (5.74,5.84)	6.98 (6.85,7.11)	<0.0001
Triglyceride, mmol/L	0.62 (0.61,0.63)	1.01 (1.01,1.02)	1.48 (1.47,1.49)	2.82 (2.74,2.91)	<0.0001
Total cholesterol, mmol/L	4.52 (4.48,4.57)	4.94 (4.88,4.99)	5.16 (5.11,5.21)	5.49 (5.44,5.55)	<0.0001
LDL-C, mmol/L	2.58 (2.55,2.62)	3.00 (2.95,3.04)	3.15 (3.11,3.20)	3.13 (3.08,3.18)	<0.0001
HDL-C, mmol/L	1.66 (1.63,1.68)	1.47 (1.45,1.49)	1.33 (1.31,1.34)	1.14 (1.12,1.16)	<0.0001
Gender, %					<0.0001
Male	39.51 (37.04,42.03)	48.90 (46.82,50.99)	52.91 (50.65,55.16)	57.22 (55.01,59.40)	
Female	60.49 (57.97,62.96)	51.10 (49.01,53.18)	47.09 (44.84,49.35)	42.78 (40.60,44.99)	
Races, %					<0.0001
Mexican American	6.09 (4.89,7.58)	7.62 (6.26,9.25)	8.75 (7.06,10.79)	10.50 (8.66,12.68)	
Other Hispanic	4.17 (3.19,5.43)	4.55 (3.66,5.64)	5.37 (4.33,6.63)	4.66 (3.63,5.96)	
Non-Hispanic White	67.61 (64.42,70.64)	70.79 (67.82,73.59)	70.52 (67.33,73.53)	72.72 (69.50,75.73)	
Non-Hispanic Black	15.11 (13.10,17.37)	10.83 (9.44,12.40)	7.62 (6.45,8.98)	5.81 (4.88,6.90)	
Other Race	7.02 (5.89,8.34)	6.21 (5.15,7.47)	7.74 (6.65,8.99)	6.31 (5.17,7.69)	
Married or with Partner	62.22 (59.65,64.72)	65.75 (63.21,68.20)	66.27 (63.85,68.61)	67.40 (64.81,69.89)	0.0063
PIR, %					0.0093
< 1	12.47 (10.83,14.33)	12.92 (11.24,14.81)	12.83 (11.26,14.58)	12.89 (11.48,14.44)	
1–3	33.34 (31.03,35.74)	35.27 (32.22,38.44)	38.08 (35.21,41.03)	38.98 (36.54,41.48)	
> 3	54.18 (51.28,57.06)	51.81 (48.32,55.28)	49.09 (45.90,52.29)	48.13 (45.33,50.94)	
Drink, %	73.07 (70.60,75.42)	74.17 (71.49,76.68)	73.39 (70.71,75.91)	69.81 (67.20,72.29)	0.0201
Smoke, %	39.71 (36.76,42.75)	46.19 (43.14,49.27)	50.23 (47.53,52.93)	54.68 (52.26,57.08)	<0.0001
diabetes, %					<0.0001
Yes	2.20 (1.66,2.90)	4.42 (3.60,5.42)	7.62 (6.38,9.08)	23.66 (21.69,25.76)	
No	96.79 (95.95,97.46)	93.82 (92.63,94.82)	89.10 (87.26,90.71)	73.82 (71.71,75.83)	
Borderline	1.01 (0.66,1.56)	1.76 (1.21,2.55)	3.28 (2.43,4.41)	2.51 (1.86,3.38)	
Hypertension, %	18.58 (16.73,20.57)	29.49 (27.26,31.82)	37.62 (35.40,39.89)	45.80 (43.23,48.39)	<0.0001
cancer, %	7.40 (6.12,8.92)	10.66 (9.35,12.13)	10.01 (8.69,11.49)	12.64 (11.17,14.27)	<0.0001
CKD, %	9.29 (7.97,10.79)	12.67 (11.06,14.47)	15.17 (13.43,17.08)	21.62 (19.82,23.53)	<0.0001
CVD, %	4.97 (4.15,5.94)	7.74 (6.60,9.06)	9.66 (8.31,11.20)	13.48 (11.88,15.27)	<0.0001
CRS, %	1.86 (1.41,2.45)	3.07 (2.50,3.77)	3.92 (3.20,4.79)	6.03 (5.08,7.15)	<0.0001

Continuous variables were listed as weighted mean (95% CI), P-value was by survey-weighted linear regression. Categorical variables were listed as weighted percentage (95% CI), P-value was by survey-weighted Chi-square test. LDL-C, Low-density lipoprotein cholesterol; HDL-C, High-density lipoprotein cholesterol; BMI, body mass index; PIR family income-poverty ratio; CKD, chronic kidney disease; CVD, cardiovascular disease; CRS, cardiorenal syndrome.

### TyG and its composite obesity indexes in relation to CKD, CVD, and CRS

**Table 2** illustrates the connection between TyG and its composite obesity indexes with CKD, CVD, and CRS. Supplemental files: **Supplementary Tables S4–S6** provide comprehensive data on each association. TyG and its composite obesity indicators were positively linked with the likelihood of developing CKD, CVD, and CRS in the fully adjusted model (Model 3). TyG exhibited the strongest positive association with CKD (OR 1.42, 95% CI 1.11, 1.82; P = 0.007) (**Table 2**), followed by TyG-WHtR (HR 1.39, 95% CI 1.08, 1.78; P = 0.011) (**Supplementary Table S4**), based on further analysis utilizing TyG and its composite obesity indicators as stratification variables (quartiles). TyG-WHtR showed the best correlation with CVD (OR 1.63, 95% CI 1.19, 2.22; P = 0.003) (**Supplementary Table S4**), followed by TyG-WC (OR 1.57, 95% CI 1.14, 2.18; P = 0.008) (**Supplementary Table S6**). CRS was best correlated with TyG-WHtR (OR 1.44, 95% CI 1.00, 2.08; P = 0.055) (**Supplementary Table S4**).

**Table 2:** The association between TyG index and the risk of CKD, CVD and CRS.

Exposure	Model I		Model II		Model III	
	OR (95%CI)	P-value	OR (95%CI)	P-value	OR (95%CI)	P-value
CKD						
TYG	1.76 (1.58, 1.96)	<0.001	1.62 (1.41, 1.86)	<0.001	1.37 (1.19, 1.59)	<0.001
Categories						
Q1	Ref		Ref		Ref	
Q2	1.42 (1.16, 1.74)	0.001	1.10 (0.87, 1.40)	0.417	1.02 (0.81, 1.30)	0.854
Q3	1.75 (1.39, 2.20)	<0.001	1.29 (0.99, 1.69)	0.068	1.11 (0.85, 1.45)	0.440
Q4	2.69 (2.19, 3.32)	<0.001	1.97 (1.53, 2.53)	<0.001	1.42 (1.11, 1.82)	0.0071
P for trend	<0.001		<0.001		0.006	
CVD						
TYG	1.71 (1.51, 1.92)	<0.001	1.49 (1.29, 1.72)	<0.001	1.30 (1.10, 1.53)	0.003
Categories						
Q1	Ref		Ref		Ref	
Q2	1.60 (1.28, 2.01)	<0.001	1.15 (0.91, 1.46)	0.250	1.10 (0.85, 1.42)	0.490
Q3	2.04 (1.60, 2.62)	<0.001	1.36 (1.04, 1.78)	0.030	1.20 (0.90, 1.59)	0.211
Q4	2.98 (2.34, 3.79)	<0.001	1.92 (1.52, 2.44)	<0.001	1.50 (1.15, 1.96)	0.004
P for trend	<0.001		<0.001		0.005	
CRS						
TYG	1.84 (1.59, 2.13) <0.001		1.74 (1.41, 2.13) <0.001		1.31 (1.06, 1.64) 0.017	
Categories						
Q1	Ref		Ref		Ref	
Q2	1.67 (1.19, 2.34)	0.004	1.11 (0.76, 1.62)	0.594	0.98 (0.67, 1.44)	0.917
Q3	2.15 (1.52, 3.02)	<0.001	1.33 (0.89, 1.98)	0.162	1.06 (0.71, 1.57)	0.788
Q4	3.38 (2.46, 4.64)	<0.001	2.10 (1.49, 2.96)	<0.001	1.28 (0.90, 1.81)	0.176
P for trend	<0.001		<0.001		0.086	

Model 1: No covariates were adjusted.

Model 2: Age, gender, and race were adjusted.

Model 3: Age, gender, race, marital.status, PIR, smoking, drinking, cancer, hypertension, diabetes and cholesterol were adjusted.

The variables adjusted in each model were the factors mentioned above except the stratification variables.

Data were listed as the weighted hazard ratio estimates and 95% confidence intervals, with Q, quintile; Ref, reference.

Tests for trends based on the variables containing the median values for each quartile.

#### RCS analysis

**Figure 2:**
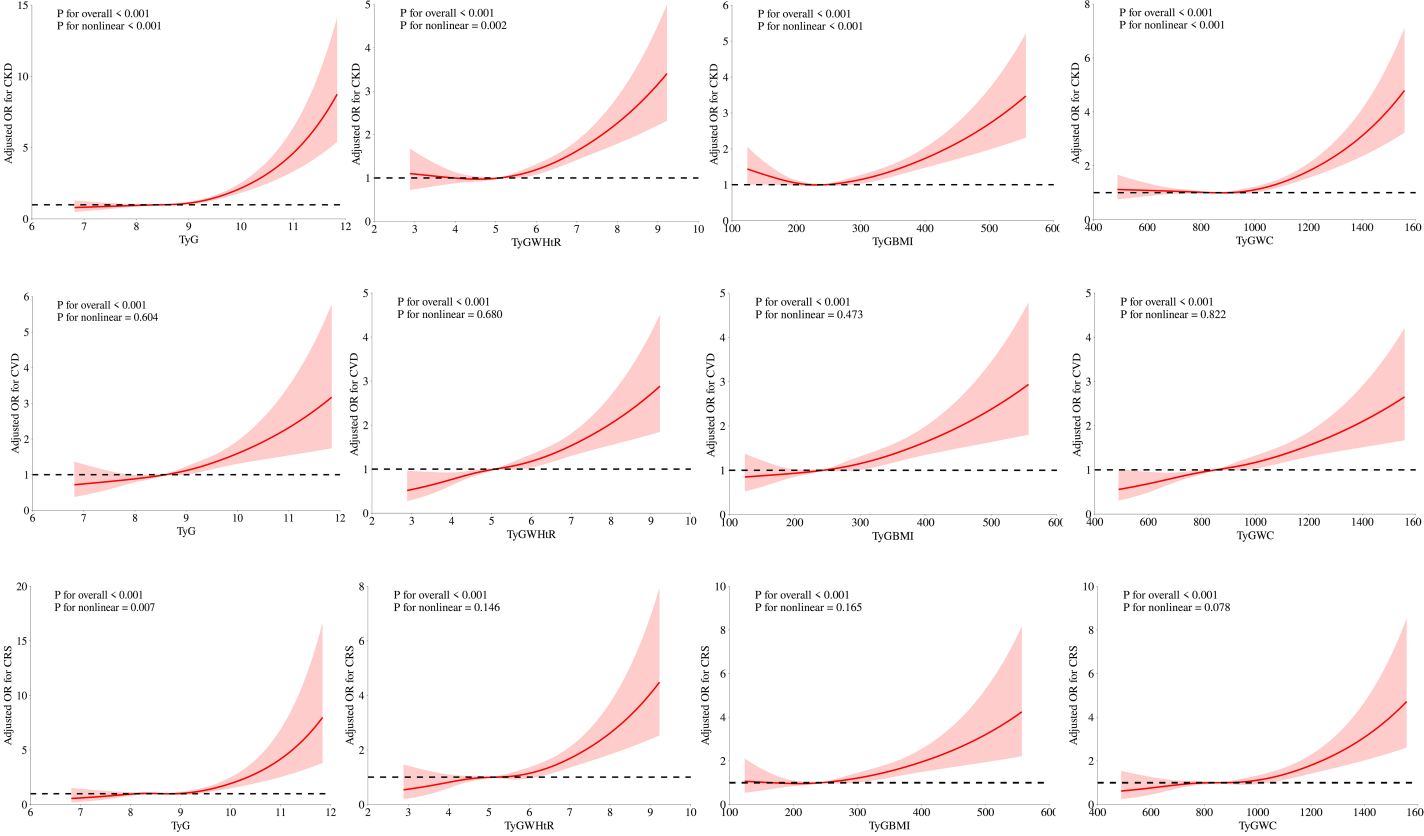
Associations between TyG, TyG-WHtR, TyG-BMI, and TyG-WC with CKD, CVD, and CRS were evaluated by RCS after adjustment for the covariables.

**Figure 2** illustrates how we employed RCS curves to dynamically construct and visualize potential connections between TyG and its composite indexes and the risk of CKD, CVD, and CRS. After controlling for all factors in the primary analytic model (Model 3), TyG and CVD exhibited a steady linear positive connection. TyG-WC, TyG-WHtR, and TyG-BMI all demonstrated a substantial and linearly positive correlation with CVD and CRS (P for overall < 0.001, P for nonlinear > 0.05). All four TyG indicators and their composite obesity indexes showed nonlinear relationships (P for overall < 0.001, P for nonlinear < 0.05) with CKD and between TyG and CRS, suggesting that TyG and its derived indices may require different analytic approaches when predicting various aspects of cardiorenal disease.

#### ROC analysis

**Figure 3:**
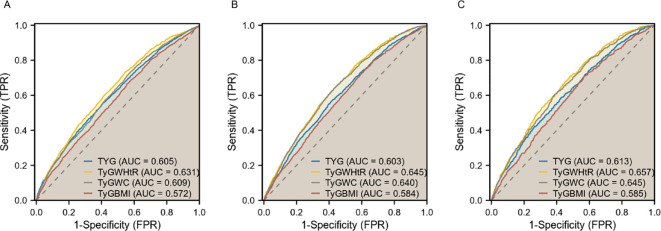
ROC curves of TyG, TyG-WHtR, TyG-WC, and TyG-BMI in relation to CKD, CVD, and CRS. **(A)** CKD; **(B)** CVD; **(C)** CRS.

In diagnosing CKD, TyG-WHtR demonstrated the highest potency with an AUC of 0.631 (95% CI 0.618-0.643), followed by TyG-WC with an AUC of 0.609 (95% CI 0.596-0.622). For diagnosing CVD, TyG-WHtR exhibited the highest diagnostic effectiveness with an AUC of 0.645 (95% CI 0.630-0.660), followed closely by TyG-WC, which had an AUC of 0.640 (95% CI 0.625-0.655). Both TyG-WHtR and TyG-WC were also superior in detecting CRS (AUC = 0.657 for TyG-WHtR, 95% CI 0.636-0.677; AUC = 0.645 for TyG-WC, 95% CI 0.624-0.666). These findings imply that TyG-WHtR and TyG-WC may serve as effective risk assessment tools for cardiac and renal disorders (**Figure 3**).

#### Subgroup analysis

**Figure 4:**
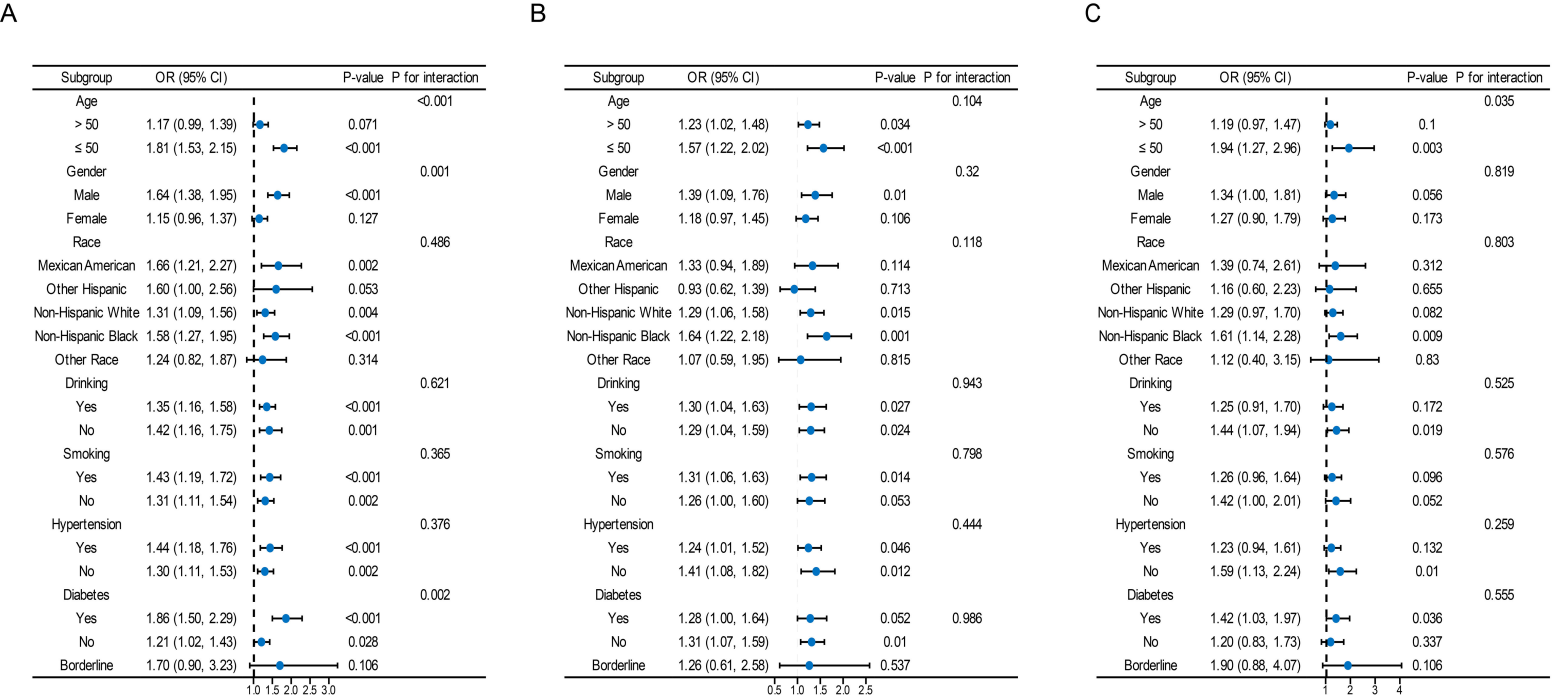
Subgroup analysis for the association between the TyG index and the risk of CKD, CVD, and CRS. The variables adjusted in each model were the factors mentioned above except the stratification variables. Data were listed as the weighted odd ratio estimates and 95% confidence intervals.

Overall, a significant interaction was found between age, sex, and diabetes mellitus in the relationship between TyG and its composite obesity indexes and the risk of CKD, with stronger associations observed in individuals aged ≤ 50, males, and diabetic patients (P for interaction < 0.05). Furthermore, there were noteworthy interplays among distinct subsets of the indicators. For instance, the detrimental impacts of TyG-WHtR, TyG-BMI, TyG-WC on CVD and CRS seemed to be more prominent in younger people, and the detrimental effects of TyG-WC on CVD and CRS seemed to be more prominent in people who consumed alcohol (**Figure 4**, **Supplementary Figures S1–S3**).

## Discussion

Our preliminary results indicate a positive association between TyG and its composite obesity indexes and the risk of developing CKD, CVD, and CRS. (1) Among the various parameters, TyG-WHtR demonstrated the strongest connection with CVD and CRS, while the highest association with CKD was found in TyG. (2) Nonlinear relationships were observed between TyG, TyG-WHtR, TyG-WC, and TyG-BMI and the probability of CKD, as well as between TyG and CRS. (3) In contrast to TyG, TyG-WHtR and TyG-WC were more effective in diagnosing CKD, CVD, and CRS. (4) The positive association between TyG and its composite obesity indexes and CKD was discovered to be more commonly in younger individuals (≤ 50), males, and those with diabetes mellitus.

TyG and its composite obesity indexes have been established as simple and reliable markers of IR and metabolic health for clinical use. They are derived from widely available measures of FBG, TG, and obesity, with research demonstrating that TyG-related obesity indicators are more effective than TyG alone in detecting IR (25, 26). Previous studies have shown a strong association between the TyG index and coronary artery stenosis, CKD, and atherosclerotic cardiovascular disease (ASCVD) in the general population, as well as among individuals with metabolic disorders such as diabetes and hyperuricemia (27, 28). For instance, a national prospective cohort study in China indicated that individuals with higher TyG levels faced an increased risk of stroke (29). Cross-sectional studies based on NHANES data have revealed that higher TyG scores are significantly associated with elevated UACR levels, albuminuria, and CKD values (30). However, the majority of these studies have concentrated on the impacts of the most recent TyGs, leaving research on TyG-related obesity indices rather scattered. As a result, it is necessary to evaluate these characteristics at the same time and compare the various indices.

Few studies, to our knowledge, have comprehensively evaluated the relationships between TyG in combination with obesity indices and CKD, CVD, and CRS. Our research indicates a positive correlation between TyG’s combined obesity metrics and the risks of CKD, CVD, and CRS, That’s consistent with previous reports. High TyG and its combined obesity markers were linked to higher risk of CVD and mortality, according to research by Dang et al. (31). In an older cohort, Kim et al. discovered that an elevated TyG index was a predictor of CKD risk (32). Additionally, a previous cross-sectional clinical investigation found that a higher TyG index was independently correlated with the severity of coronary artery disease in individuals with CKD (33). However, the positive effect sizes in Model 3 became nonsignificant as the TyG, TyG-WC, and TyG-BMI indexes increased. This finding may relate to the multidimensionality of the CRS data, the grouping strategy, and the selected modeling options.

Additionally, the ROC curves demonstrated that TyG-WHtR and TyG-WC outperformed TyG-BMI in terms of diagnostic efficacy for CKD, CVD, and CRS. A study investigating CKD in a Chinese population revealed that TyG-WHtR has higher predictive performance than TyG alone (34). In a 15-year cohort analysis, TyG-WC and TyG-WHtR were shown to be superior to TyG for predicting new ASCVD events (35). In another study, elevated baseline TyG-WC levels were reported to be associated with an increased risk of first MI, and TyG-WC has been proposed as a simple and effective marker for clinical application (36). This superiority may be attributed to the fact that WC and are key indicators of central abdominal obesity, which is associated with higher visceral fat accumulation, chronic inflammation, oxidative stress, and neuroendocrine disruption, leading to the coexistence of chronic diseases (37). Our findings establish TyG-WC and TyG-WHtR as valuable risk indicators for cardiorenal illness and suggest that they may be effective for the co-management of cardiovascular and renal disorders.

According to subgroup analyses, TyG and its composite obesity measurements were more strongly associated with cardiorenal illness in younger individuals. These age-related effects are consistent with prior investigations (38, 39). One possible explanation is because older individuals are more likely to be taking multiple medications and have a greater cardiac and renal burden beyond IR or obesity, which somewhat weakens the prediction of cardiac and renal disease risk outcomes by TyG and its obesity metrics.

The precise biological mechanisms underlying the relationship between TyG and its composite obesity indexes and cardiac and renal illness are not fully understood; however, several possible explanations exist. Elevated TyG and its composite obesity index levels are linked to IR and increased adiposity (40, 41). Abnormalities in hemodynamics and neurohumoral control are important factors in the interaction between cardiac and renal failure (42, 43). Glycolipotoxicity induced by IR can lead to oxidative stress condition, resulting in endothelial dysfunction and increased cardio-renal vascular resistance (44, 45). Persistent IR activates the sympathetic nervous system and the renin-angiotensin-aldosterone system excessively, which damages the kidneys and causes abnormalities in blood pressure, electrolyte imbalances, and cardiac workload (46, 47). Furthermore, IR and metabolic disorders such as hyperlipidemia and obesity accumulate harmful metabolites contribute to a vicious cycle of CKD, CVD, and CRS (48, 49).

This is the first study to focus on comprehensively evaluating the association between TyG- and its obesity index and heart and kidney disease. Our results support that TyG-WC, TyG-WHtR are valuable tools for predicting the risk of cardiorenal disease, which may help to reduce the cost of screening, early detection of individuals at high risk of cardiorenal disease and improve risk stratification in clinical practice. Effective interventions targeting TyG-WC, TyG-WHtR indices could be developed in the future to prevent the risk of cardiorenal disease. Focusing on TyG-WC, TyG-WHtR levels may provide more precise and personalized strategies for the prevention of cardiorenal disease.

Our study demonstrates several research strengths. Our findings highlight the potential of TyG and its composite obesity indexes in the therapeutic management of cardiac and renal disorders. First, we utilized the NHANES database, which employs a complex multi-stage probability sampling procedure, and evaluated the data with suitable NHANES sample weights, boosting the validity and adaptability. A second strength is the inclusion of a wide range of covariates, which improves accuracy. Finally, The large sample size ensures the reliability of our assessment results. However, there are limitations to this study. First, the diagnosis of cardiovascular events and certain covariates rely on self-reporting, which may introduce the possibility of measurement error and inaccurate data collection; Second, although we have adjusted for as many potential confounders as possible, the possibility that unmeasured variables or residuals of unknown confounders cause confounding effects cannot be completely ruled out; Third, the analysis examined only baseline TyG and its derivatives, and it is unclear how they affect CKD, CVD, and CRS over time; Furthermore, although CRS is divided into 5 subtypes, our study sample represents only chronic CRS, and we were unable to determine the order of decline in cardiovascular and renal functions; Finally, our study was conducted based on a U.S. population, and the applicability of the findings may vary regionally, necessitating further exploration for validation in a broader population.

## Conclusions

In conclusion, TyG-WC and TyG-WtHR were positively associated with heart and kidney disease in the US population, and showed higher predictive and diagnostic efficacy compared to TyG. Future applications in public health services have the potential to make them effective markers for identifying cardio-renal disease risk, early prevention and co-management of cardio-renal disease.

## Data Availability

Publicly available datasets were analyzed in this study. This data can be found here: https://www.cdc.gov/nchs/nhanes/index.htm.
