# Supplementary material for: Association between triglyceride-glucose index and its composite obesity indexes and cardio-renal disease: analysis of the NHANES 2013-2018 cycle
Source: Front Endocrinol (Lausanne). 2025 Jan 31;16:1505808. doi: 10.3389/fendo.2025.1505808 (PMC11825323; doi:10.3389/fendo.2025.1505808)
Supplement: Supplementary file 1 [file DataSheet1.docx]

Supplementary Table 1.

Baseline characteristics according to triglyceride-glucose-waist-to-height ratio (TyG-WtHR) quartiles: NHANES, 2003-2018

|  | Triglyceride-glucose-waist-to-height ratio (TyG-WtHR) (N = 11,491) | | | | |
| --- | --- | --- | --- | --- | --- |
|  | Q1(2.58–4.39) | Q2(4.39–5.09) | Q3(5.09–5.84) | Q4(5.84–10.64) | P-value |
| Age, years | 40.48 (39.60 ,41.35) | 48.57 (47.78 ,49.35) | 50.59 (49.74 ,51.43) | 52.10 (51.26 ,52.94) | <0.0001 |
| Height, cm | 169.98 (169.54 ,170.43) | 169.86 (169.38 ,170.34) | 169.07 (168.55 ,169.60) | 167.42 (166.91 ,167.93) | <0.0001 |
| BMI, kg/m2 | 22.78 (22.63 ,22.94) | 27.04 (26.87 ,27.22) | 30.58 (30.40 ,30.76) | 37.42 (37.06 ,37.78) | <0.0001 |
| Waist circumference, cm | 82.33 (81.94 ,82.71) | 95.22 (94.80 ,95.64) | 104.53 (104.12 ,104.95) | 120.29 (119.64 ,120.95) | <0.0001 |
| Fasting glucose, mmol/L | 5.22 (5.18 ,5.25) | 5.56 (5.52 ,5.60) | 5.93 (5.85 ,6.01) | 6.86 (6.75 ,6.96) | <0.0001 |
| Triglyceride, mmol/L | 0.82 (0.80 ,0.84) | 1.20 (1.17 ,1.23) | 1.64 (1.59 ,1.69) | 2.24 (2.14 ,2.34) | <0.0001 |
| Total cholesterol, mmol/L | 4.69 (4.64 ,4.74) | 5.06 (5.01 ,5.12) | 5.15 (5.10 ,5.21) | 5.18 (5.11 ,5.25) | <0.0001 |
| LDL-C, mmol/L | 2.66 (2.62 ,2.71) | 3.07 (3.02 ,3.12) | 3.11 (3.07 ,3.16) | 3.01 (2.95 ,3.06) | <0.0001 |
| HDL-C, mmol/L | 1.65 (1.63 ,1.67) | 1.44 (1.42 ,1.47) | 1.30 (1.28 ,1.32) | 1.19 (1.18 ,1.21) | <0.0001 |
| Gender, % |  |  |  |  | <0.0001 |
| Male | 46.33 (44.12 ,48.56) | 53.44 (51.32 ,55.54) | 53.89 (51.76 ,56.01) | 43.52 (41.07 ,46.01) |  |
| Female | 53.67 (51.44 ,55.88) | 46.56 (44.46 ,48.68) | 46.11 (43.99 ,48.24) | 56.48 (53.99 ,58.93) |  |
| Races, % |  |  |  |  | <0.0001 |
| Mexican American | 4.95 (4.09 ,5.98) | 7.95 (6.48 ,9.71) | 10.74 (8.86 ,12.95) | 9.62 (7.74 ,11.90) |  |
| Other Hispanic | 4.36 (3.37 ,5.64) | 4.63 (3.58 ,5.98) | 4.86 (3.91 ,6.04) | 4.87 (3.94 ,6.02) |  |
| Non-Hispanic White | 70.40 (67.61 ,73.05) | 69.92 (66.88 ,72.80) | 69.48 (66.23 ,72.56) | 71.51 (68.05 ,74.74) |  |
| Non-Hispanic Black | 11.48 (9.88 ,13.31) | 9.93 (8.48 ,11.61) | 9.04 (7.81 ,10.43) | 9.43 (7.99 ,11.09) |  |
| Other Race | 8.80 (7.54 ,10.24) | 7.57 (6.21 ,9.19) | 5.88 (4.80 ,7.18) | 4.57 (3.61 ,5.76) |  |
| Married or with Partner | 61.25 (58.67 ,63.78) | 67.16 (64.51 ,69.69) | 69.58 (67.00 ,72.05) | 63.81 (61.21 ,66.33) | <0.0001 |
| PIR, % |  |  |  |  | <0.0001 |
| < 1 | 12.02 (10.61 ,13.60) | 11.70 (10.31 ,13.24) | 12.42 (11.01 ,13.97) | 15.23 (13.29 ,17.39) |  |
| 1–3 | 32.53 (29.95 ,35.21) | 33.90 (31.50 ,36.38) | 38.08 (35.68 ,40.55) | 41.68 (38.99 ,44.43) |  |
| > 3 | 55.45 (52.37 ,58.49) | 54.40 (51.72 ,57.06) | 49.50 (46.75 ,52.26) | 43.09 (39.97 ,46.26) |  |
| Drink, % | 77.76 (75.47 ,79.90) | 74.66 (72.09 ,77.06) | 72.43 (69.44 ,75.23) | 64.52 (61.77 ,67.18) | <0.0001 |
| Smoke, % | 43.48 (40.62 ,46.38) | 45.91 (43.16 ,48.70) | 49.84 (47.02 ,52.65) | 51.30 (48.95 ,53.65) | <0.0001 |
| diabetes, % |  |  |  |  | <0.0001 |
| Yes | 1.40 (0.99 ,1.97) | 4.35 (3.59 ,5.25) | 9.13 (7.78 ,10.69) | 23.62 (21.67 ,25.69) |  |
| No | 98.13 (97.41 ,98.66) | 93.72 (92.44 ,94.78) | 88.45 (86.75 ,89.96) | 72.44 (70.43 ,74.37) |  |
| Borderline | 0.47 (0.26 ,0.84) | 1.94 (1.39 ,2.71) | 2.42 (1.72 ,3.37) | 3.94 (3.21 ,4.82) |  |
| Hypertension, % | 13.25 (11.57 ,15.13) | 29.78 (27.62 ,32.04) | 38.24 (35.95 ,40.57) | 52.24 (49.87 ,54.61) | <0.0001 |
| cancer, % | 7.29 (6.14 ,8.63) | 10.11 (8.74 ,11.67) | 11.18 (9.76 ,12.79) | 12.33 (10.72 ,14.15) | <0.0001 |
| CKD, % | 7.44 (6.34 ,8.72) | 13.54 (12.02 ,15.22) | 14.99 (13.26 ,16.90) | 23.43 (21.43 ,25.55) | <0.0001 |
| CVD, % | 3.67 (2.92 ,4.61) | 7.83 (6.59 ,9.28) | 9.69 (8.43 ,11.12) | 15.18 (13.48 ,17.05) | <0.0001 |
| CRS, % | 1.30 (0.94 ,1.79) | 2.76 (2.23 ,3.42) | 4.05 (3.32 ,4.94) | 7.04 (6.07 ,8.15) | <0.0001 |

Continuous variables were listed as weighted mean (95% CI), P-value was by survey-weighted linear regression. Categorical variables were listed as weighted percentage (95% CI), P-value was by survey-weighted Chi-square test.

Supplementary Table 2. Baseline characteristics according to triglyceride-glucose-body mass index (TyG-BMI) quartiles: NHANES, 2003-2018

| Triglyceride-glucose-body mass index (TyG-BMI) (N = 11,491) | | | | | |
| --- | --- | --- | --- | --- | --- |
|  | Q1(113.51–204.97) | Q2(204.97–243.41) | Q3(243.41–289.76) | Q4(289.76–679.46) | P-value |
| Age, years | 43.44 (42.44 ,44.44) | 49.33 (48.48 ,50.17) | 49.46 (48.57 ,50.35) | 48.59 (47.86 ,49.32) | <0.0001 |
| Height, cm | 168.52 (168.02 ,169.01) | 169.72 (169.26 ,170.18) | 169.54 (169.04 ,170.04) | 168.88 (168.43 ,169.34) | 0.0006 |
| BMI, kg/m2 | 22.12 (22.01 ,22.22) | 26.47 (26.39 ,26.55) | 30.26 (30.13 ,30.39) | 38.23 (37.91 ,38.54) | <0.0001 |
| Waist circumference, cm | 81.97 (81.61 ,82.33) | 94.57 (94.20 ,94.93) | 103.70 (103.28 ,104.12) | 120.05 (119.41 ,120.70) | <0.0001 |
| Fasting glucose, mmol/L | 5.26 (5.22 ,5.29) | 5.66 (5.59 ,5.73) | 5.88 (5.83 ,5.94) | 6.66 (6.56 ,6.76) | <0.0001 |
| Triglyceride, mmol/L | 0.85 (0.83 ,0.88) | 1.24 (1.19 ,1.29) | 1.63 (1.58 ,1.68) | 2.08 (1.99 ,2.17) | <0.0001 |
| Total cholesterol, mmol/L | 4.74 (4.69 ,4.79) | 5.06 (5.00 ,5.12) | 5.14 (5.09 ,5.20) | 5.11 (5.04 ,5.17) | <0.0001 |
| LDL-C, mmol/L | 2.68 (2.64 ,2.72) | 3.04 (2.99 ,3.09) | 3.11 (3.07 ,3.15) | 3.00 (2.95 ,3.05) | <0.0001 |
| HDL-C, mmol/L | 1.67 (1.65 ,1.69) | 1.46 (1.43 ,1.48) | 1.29 (1.27 ,1.30) | 1.20 (1.18 ,1.21) | <0.0001 |
| Gender, % |  |  |  |  | <0.0001 |
| Male | 41.13 (38.74 ,43.55) | 54.64 (52.33 ,56.92) | 54.87 (52.70 ,57.02) | 47.37 (45.00 ,49.75) |  |
| Female | 58.87 (56.45 ,61.26) | 45.36 (43.08 ,47.67) | 45.13 (42.98 ,47.30) | 52.63 (50.25 ,55.00) |  |
| Races, % |  |  |  |  | <0.0001 |
| Mexican American | 5.31 (4.46 ,6.32) | 8.02 (6.52 ,9.83) | 10.12 (8.25 ,12.35) | 9.49 (7.78 ,11.52) |  |
| Other Hispanic | 3.93 (2.97 ,5.19) | 4.90 (3.81 ,6.29) | 4.92 (3.90 ,6.17) | 5.00 (4.04 ,6.17) |  |
| Non-Hispanic White | 71.81 (69.16 ,74.33) | 70.39 (67.29 ,73.31) | 69.67 (66.25 ,72.90) | 69.23 (65.92 ,72.35) |  |
| Non-Hispanic Black | 9.12 (7.77 ,10.67) | 9.23 (7.86 ,10.80) | 10.17 (8.78 ,11.77) | 11.75 (10.08 ,13.65) |  |
| Other Race | 9.82 (8.47 ,11.36) | 7.46 (6.25 ,8.88) | 5.12 (4.06 ,6.44) | 4.54 (3.65 ,5.64) |  |
| Married or with Partner | 60.89 (58.19 ,63.53) | 66.23 (63.33 ,69.01) | 68.46 (65.72 ,71.08) | 66.15 (63.50 ,68.70) | 0.0002 |
| PIR, % |  |  |  |  | <0.0001 |
| < 1 | 12.28 (10.64 ,14.13) | 12.00 (10.70 ,13.43) | 12.20 (10.80 ,13.74) | 14.66 (12.85 ,16.68) |  |
| 1–3 | 33.42 (30.67 ,36.29) | 35.70 (33.37 ,38.09) | 36.89 (34.54 ,39.31) | 39.49 (36.70 ,42.35) |  |
| > 3 | 54.30 (50.92 ,57.64) | 52.30 (49.70 ,54.89) | 50.91 (48.05 ,53.77) | 45.85 (42.87 ,48.86) |  |
| Drink, % | 74.58 (72.22 ,76.81) | 75.54 (73.23 ,77.71) | 73.39 (70.55 ,76.05) | 66.95 (63.93 ,69.82) | <0.0001 |
| Smoke, % | 44.87 (41.84 ,47.94) | 46.94 (44.34 ,49.56) | 49.48 (46.86 ,52.11) | 48.59 (46.09 ,51.09) | 0.0353 |
| diabetes, % |  |  |  |  | <0.0001 |
| Yes | 1.88 (1.39 ,2.54) | 6.17 (5.08 ,7.48) | 9.22 (7.92 ,10.71) | 19.87 (18.21 ,21.64) |  |
| No | 97.01 (96.14 ,97.69) | 92.28 (90.76 ,93.57) | 88.41 (86.84 ,89.82) | 76.65 (74.89 ,78.32) |  |
| Borderline | 1.11 (0.74 ,1.65) | 1.55 (1.04 ,2.30) | 2.37 (1.74 ,3.21) | 3.48 (2.83 ,4.28) |  |
| Hypertension, % | 15.97 (14.43 ,17.63) | 30.33 (28.19 ,32.56) | 36.27 (33.84 ,38.78) | 48.52 (46.06 ,50.98) | <0.0001 |
| cancer, % | 9.59 (8.17 ,11.21) | 10.16 (9.08 ,11.35) | 10.15 (8.82 ,11.66) | 10.50 (9.14 ,12.03) | 0.8036 |
| CKD, % | 10.27 (9.03 ,11.65) | 14.55 (12.83 ,16.47) | 14.02 (12.52 ,15.67) | 19.40 (17.66 ,21.26) | <0.0001 |
| CVD, % | 5.68 (4.72 ,6.82) | 8.51 (7.20 ,10.05) | 9.67 (8.34 ,11.18) | 11.67 (10.28 ,13.21) | <0.0001 |
| CRS, % | 2.31 (1.83 ,2.91) | 3.27 (2.65 ,4.04) | 3.93 (3.23 ,4.77) | 5.20 (4.41 ,6.11) | <0.0001 |

Continuous variables were listed as weighted mean (95% CI), P-value was by survey-weighted linear regression. Categorical variables were listed as weighted percentage (95% CI), P-value was by survey-weighted Chi-square test.

Supplementary Table 3. Baseline characteristics according to triglyceride-glucose-waist circumference (TyG-WC) quartiles: NHANES, 2003 - 2018

| Triglyceride-glucose-waist circumference (TyG-WC) (N = 11,491) | | | | | |
| --- | --- | --- | --- | --- | --- |
|  | Q1(453.12–734.63) | Q2(734.63–852.15) | Q3(852.15–976.06) | Q4(976.06–1697.36) | P-value |
| Age, years | 41.24 (40.30 ,42.19) | 48.31 (47.47 ,49.14) | 50.56 (49.76 ,51.37) | 50.95 (50.18 ,51.73) | <0.0001 |
| Height, cm | 167.00 (166.49 ,167.50) | 168.56 (168.05 ,169.07) | 170.06 (169.55 ,170.57) | 171.19 (170.73 ,171.65) | <0.0001 |
| BMI, kg/m2 | 22.68 (22.52 ,22.84) | 26.99 (26.85 ,27.13) | 30.29 (30.11 ,30.46) | 36.92 (36.56 ,37.28) | <0.0001 |
| Waist circumference, cm | 81.24 (80.89 ,81.58) | 94.26 (93.99 ,94.53) | 103.98 (103.68 ,104.28) | 120.56 (119.89 ,121.22) | <0.0001 |
| Fasting glucose, mmol/L | 5.19 (5.16 ,5.22) | 5.55 (5.51 ,5.60) | 5.90 (5.83 ,5.98) | 6.80 (6.70 ,6.90) | <0.0001 |
| Triglyceride, mmol/L | 0.82 (0.80 ,0.84) | 1.18 (1.15 ,1.21) | 1.55 (1.51 ,1.59) | 2.25 (2.15 ,2.35) | <0.0001 |
| Total cholesterol, mmol/L | 4.71 (4.67 ,4.76) | 5.05 (4.99 ,5.11) | 5.13 (5.08 ,5.18) | 5.16 (5.09 ,5.23) | <0.0001 |
| LDL-C, mmol/L | 2.66 (2.62 ,2.70) | 3.05 (3.00 ,3.10) | 3.12 (3.08 ,3.16) | 3.01 (2.96 ,3.06) | <0.0001 |
| HDL-C, mmol/L | 1.68 (1.66 ,1.71) | 1.46 (1.44 ,1.48) | 1.30 (1.28 ,1.32) | 1.17 (1.15 ,1.18) | <0.0001 |
| Gender, % |  |  |  |  | <0.0001 |
| Male | 35.18 (32.82 ,37.62) | 49.38 (46.98 ,51.79) | 56.78 (54.71 ,58.82) | 57.33 (54.99 ,59.64) |  |
| Female | 64.82 (62.38 ,67.18) | 50.62 (48.21 ,53.02) | 43.22 (41.18 ,45.29) | 42.67 (40.36 ,45.01) |  |
| Races, % |  |  |  |  | <0.0001 |
| Mexican American | 5.98 (4.98 ,7.17) | 8.74 (7.11 ,10.70) | 10.01 (8.15 ,12.24) | 8.16 (6.52 ,10.15) |  |
| Other Hispanic | 4.55 (3.52 ,5.87) | 5.45 (4.21 ,7.04) | 4.53 (3.55 ,5.76) | 4.16 (3.29 ,5.24) |  |
| Non-Hispanic White | 69.02 (66.12 ,71.77) | 66.87 (63.58 ,70.00) | 71.32 (67.82 ,74.58) | 74.15 (71.11 ,76.98) |  |
| Non-Hispanic Black | 10.93 (9.40 ,12.67) | 10.70 (9.24 ,12.36) | 9.29 (7.82 ,11.00) | 9.15 (7.79 ,10.72) |  |
| Other Race | 9.52 (8.31 ,10.88) | 8.24 (6.81 ,9.95) | 4.85 (3.89 ,6.04) | 4.39 (3.51 ,5.47) |  |
| Married or with Partner | 59.61 (57.19 ,61.99) | 66.69 (64.11 ,69.17) | 68.76 (66.09 ,71.32) | 66.85 (64.21 ,69.38) | <0.0001 |
| PIR, % |  |  |  |  | 0.0046 |
| < 1 | 12.54 (11.02 ,14.23) | 12.92 (11.49 ,14.48) | 12.26 (10.83 ,13.86) | 13.37 (11.50 ,15.50) |  |
| 1–3 | 33.65 (31.01 ,36.40) | 35.06 (32.51 ,37.69) | 37.61 (35.15 ,40.14) | 39.12 (36.40 ,41.91) |  |
| > 3 | 53.81 (50.74 ,56.86) | 52.03 (49.24 ,54.81) | 50.13 (47.33 ,52.92) | 47.50 (44.38 ,50.64) |  |
| Drink, % | 74.83 (72.48 ,77.05) | 73.55 (71.13 ,75.84) | 73.16 (70.25 ,75.87) | 68.97 (66.12 ,71.69) | 0.0005 |
| Smoke, % | 41.37 (38.66 ,44.14) | 45.34 (42.77 ,47.94) | 50.21 (47.66 ,52.75) | 53.28 (50.87 ,55.68) | <0.0001 |
| diabetes, % |  |  |  |  | <0.0001 |
| Yes | 1.35 (0.95 ,1.92) | 4.59 (3.83 ,5.49) | 9.41 (8.07 ,10.93) | 21.61 (19.90 ,23.43) |  |
| No | 97.99 (97.32 ,98.50) | 93.65 (92.41 ,94.69) | 88.33 (86.66 ,89.82) | 74.55 (72.76 ,76.26) |  |
| Borderline | 0.66 (0.38 ,1.12) | 1.77 (1.22 ,2.56) | 2.26 (1.62 ,3.15) | 3.84 (3.10 ,4.76) |  |
| Hypertension, % | 14.56 (12.91 ,16.39) | 28.32 (26.39 ,30.33) | 38.09 (35.74 ,40.49) | 50.04 (47.23 ,52.84) | <0.0001 |
| cancer, % | 7.85 (6.60 ,9.32) | 9.66 (8.41 ,11.08) | 11.23 (9.86 ,12.76) | 11.84 (10.31 ,13.56) | 0.0002 |
| CKD, % | 8.82 (7.60 ,10.21) | 14.18 (12.82 ,15.66) | 14.60 (12.90 ,16.48) | 20.71 (18.79 ,22.76) | <0.0001 |
| CVD, % | 3.98 (3.14 ,5.03) | 7.65 (6.48 ,9.03) | 9.78 (8.37 ,11.40) | 14.22 (12.46 ,16.19) | <0.0001 |
| CRS, % | 1.60 (1.17 ,2.20) | 3.11 (2.51 ,3.85) | 3.86 (3.20 ,4.65) | 6.17 (5.28 ,7.21) | <0.0001 |

Continuous variables were listed as weighted mean (95% CI), P-value was by survey-weighted linear regression. Categorical variables were listed as weighted percentage (95% CI), P-value was by survey-weighted Chi-square test.

Supplementary Table 4.The association between TyG-WtHR and the risk of CKD, CVD and CRS

| Exposure | Model I | Model II | Model III |
| --- | --- | --- | --- |
|  | OR (95%CI) P-value | OR (95%CI) P-value | OR (95%CI) P-value |
| CKD |  |  |  |
| TYGWHTR | 1.54 (1.44, 1.65) <0.001 | 1.38 (1.26, 1.50) <0.001 | 1.15 (1.05, 1.26) 0.003 |
| Categories |  |  |  |
| Q1 | Ref | Ref | Ref |
| Q2 | 1.95 (1.57, 2.42) <0.001 | 1.26 (0.99, 1.60) 0.060 | 1.12 (0.90, 1.41) 0.3201 |
| Q3 | 2.19 (1.76, 2.73) <0.001 | 1.29 (1.01, 1.65) 0.043 | 1.03 (0.81, 1.30) 0.8217 |
| Q4 | 3.80 (3.09, 4.69) <0.001 | 2.22 (1.74, 2.85) <0.001 | 1.39 (1.08, 1.78) 0.0113 |
| P for trend | <0.001 | <0.001 | 0.015 |
| CVD |  |  |  |
| TYGWHTR | 1.59 (1.48, 1.71) <0.001 | 1.48 (1.35, 1.61) <0.001 | 1.21 (1.10, 1.34) <0.001 |
| Categories |  |  |  |
| Q1 | Ref | Ref | Ref |
| Q2 | 2.23 (1.66, 2.99) <0.001 | 1.33 (0.97, 1.83) 0.085 | 1.19 (0.86, 1.65) 0.298 |
| Q3 | 2.81 (2.20, 3.60) <0.001 | 1.56 (1.21, 2.01) <0.001 | 1.22 (0.92, 1.62) 0.169 |
| Q4 | 4.69 (3.60, 6.11) <0.001 | 2.72 (2.06, 3.59) <0.001 | 1.63 (1.19, 2.22) 0.003 |
| P for trend | <0.001 | <0.001 | 0.002 |
| CRS |  |  |  |
| TYGWHTR | 1.70 (1.58, 1.84) <0.001 | 1.67 (1.50, 1.85) <0.001 | 1.29 (1.15, 1.44) <0.001 |
| Categories |  |  |  |
| Q1 | Ref | Ref | Ref |
| Q2 | 2.16 (1.49, 3.12) 0.0001 | 1.14 (0.76, 1.70) 0.521 | 0.90 (0.60, 1.35) 0.610 |
| Q3 | 3.22 (2.16, 4.78) <0.001 | 1.56 (1.02, 2.37) 0.042 | 1.05 (0.68, 1.62) 0.814 |
| Q4 | 5.77 (4.15, 8.02) <0.001 | 3.01 (2.13, 4.25) <0.001 | 1.44 (1.00, 2.08) 0.055 |
| P for trend | <0.001 | <0.001 | 0.003 |

Model 1: No covariates were adjusted

Model 2: Age, gender, and race were adjusted

Model 3: Age, gender, race, marital.status, PIR, smoking, drinking, cancer, hypertension, diabetes and cholesterol were adjusted

The variables adjusted in each model were the factors mentioned above except the stratification variables.

Data were listed as the weighted hazard ratio estimates and 95% confidence intervals, with

Q, quintile; Ref, reference.

Tests for trends based on the variables containing the median values for each quartile

Supplementary Table 5.The association between TyG-BMI and the risk of CKD, CVD and CRS

| Exposure | Model I | Model II | Model III |
| --- | --- | --- | --- |
|  | OR (95%CI) P-value | OR (95%CI) P-value | OR (95%CI) P-value |
| CKD |  |  |  |
| TYGBMI | 1.47 (1.34, 1.62) <0.001 | 1.43 (1.27, 1.60) <0.001 | 1.07 (0.95, 1.21) 0.265 |
| Categories |  |  |  |
| Q1 | Ref | Ref | Ref |
| Q2 | 1.49 (1.21, 1.83) 0.0003 | 1.24 (1.01, 1.53) 0.046 | 0.98 (0.79, 1.20) 0.827 |
| Q3 | 1.43 (1.19, 1.71) 0.0003 | 1.18 (0.97, 1.45) 0.109 | 0.86 (0.70, 1.06) 0.163 |
| Q4 | 2.10 (1.76, 2.52) <0.0001 | 1.80 (1.47, 2.19) <0.001 | 1.17 (0.93, 1.46) 0.176 |
| P for trend | <0.001 | <0.001 | 0.241 |
| CVD |  |  |  |
| TYGBMI | 1.49 (1.33, 1.67) <0.001 | 1.66 (1.46, 1.90) <0.001 | 1.26 (1.09, 1.45) 0.003 |
| Categories |  |  |  |
| Q1 | Ref | Ref | Ref |
| Q2 | 1.55 (1.21, 1.98) <0.001 | 1.07 (0.82, 1.39) 0.624 | 0.95 (0.73, 1.24) 0.719 |
| Q3 | 1.78 (1.41, 2.24) <0.001 | 1.36 (1.07, 1.73) 0.013 | 1.05 (0.80, 1.39) 0.710 |
| Q4 | 2.19 (1.71, 2.81) <0.001 | 2.00 (1.55, 2.57) <0.001 | 1.21 (0.92, 1.59) 0.184 |
| P for trend | <0.001 | <0.001 | 0.123 |
| CRS |  |  |  |
| TYGBMI | 1.56 (1.38, 1.76) <0.001 | 1.66 (1.46, 1.90) <0.001 | 1.39 (1.17, 1.66) <0.001 |
| Categories |  |  |  |
| Q1 | Ref | Ref | Ref |
| Q2 | 1.43 (1.04, 1.97) 0.030 | 0.95 (0.68, 1.34) 0.784 | 0.77 (0.55, 1.09) 0.149 |
| Q3 | 1.73 (1.32, 2.28) <0.001 | 1.35 (0.99, 1.84) 0.062 | 0.91 (0.64, 1.28) 0.577 |
| Q4 | 2.32 (1.74, 3.08) <0.001 | 2.37 (1.76, 3.18) <0.001 | 1.21 (0.87, 1.66) 0.261 |
| P for trend | <0.001 | <0.001 | 0.071 |

Model 1: No covariates were adjusted

Model 2: Age, gender, and race were adjusted

Model 3: Age, gender, race, marital.status, PIR, smoking, drinking, cancer, hypertension, diabetes and cholesterol were adjusted

The variables adjusted in each model were the factors mentioned above except the stratification variables.

Data were listed as the weighted hazard ratio estimates and 95% confidence intervals, with

Q, quintile; Ref, reference.

Tests for trends based on the variables containing the median values for each quartile

Supplementary Table 6. The association between TyG-WC and the risk of CKD, CVD and CRS

| Exposure | Model I | Model II | Model III |
| --- | --- | --- | --- |
|  | OR (95%CI) P-value | OR (95%CI) P-value | OR (95%CI) P-value |
| CKD |  |  |  |
| TYGWC | 1.23 (1.18, 1.28) <0.001 | 1.20 (1.14, 1.26) <0.001 | 1.08 (1.02, 1.14) 0.006 |
| Categories |  |  |  |
| Q1 | Ref | Ref | Ref |
| Q2 | 1.71 (1.41, 2.07) <0.0001 | 1.20 (0.97, 1.48) 0.092 | 1.09 (0.88, 1.34) 0.428 |
| Q3 | 1.77 (1.42, 2.19) <0.0001 | 1.16 (0.91, 1.47) 0.233 | 0.91 (0.71, 1.16) 0.438 |
| Q4 | 2.70 (2.21, 3.30) <0.0001 | 1.93 (1.52, 2.45) <0.001 | 1.22 (0.96, 1.56) 0.106 |
| P for trend | <0.001 | <0.001 | 0.202 |
| CVD |  |  |  |
| TYGWC | 1.29 (1.23, 1.34) <0.001 | 1.25 (1.19, 1.32) <0.001 | 1.11 (1.05, 1.18) <0.001 |
| Categories |  |  |  |
| Q1 | Ref | Ref | Ref |
| Q2 | 2.00 (1.47, 2.72) <0.001 | 1.27 (0.93, 1.74) 0.131 | 1.18 (0.86, 1.62) 0.313 |
| Q3 | 2.62 (2.01, 3.41) <0.001 | 1.55 (1.19, 2.03) 0.002 | 1.22 (0.92, 1.62) 0.178 |
| Q4 | 4.00 (3.00, 5.34) <0.001 | 2.56 (1.92, 3.40) <0.001 | 1.57 (1.14, 2.18) 0.008 |
| P for trend | <0.001 | <0.001 | 0.005 |
| CRS |  |  |  |
| TYGWC | 1.32 (1.26, 1.38) <0.001 | 1.34 (1.26, 1.44) <0.001 | 1.15 (1.07, 1.23) 0.003 |
| Categories |  |  |  |
| Q1 | Ref | Ref | Ref |
| Q2 | 1.97 (1.33, 2.93) 0.001 | 1.16 (0.77, 1.73) 0.487 | 0.99 (0.66, 1.50) 0.979 |
| Q3 | 2.47 (1.72, 3.54) <0.001 | 1.35 (0.94, 1.95) 0.107 | 0.92 (0.64, 1.34) 0.681 |
| Q4 | 4.04 (2.86, 5.72) <0.001 | 2.55 (1.81, 3.60) <0.001 | 1.28 (0.89, 1.84) 0.194 |
| P for trend | <0.001 | <0.001 | 0.083 |

Model 1: No covariates were adjusted

Model 2: Age, gender, and race were adjusted

Model 3: Age, gender, race, marital.status, PIR, smoking, drinking, cancer, hypertension, diabetes and cholesterol were adjusted

The variables adjusted in each model were the factors mentioned above except the stratification variables.

Data were listed as the weighted hazard ratio estimates and 95% confidence intervals, with

Q, quintile; Ref, reference.

Tests for trends based on the variables containing the median values for each quartile


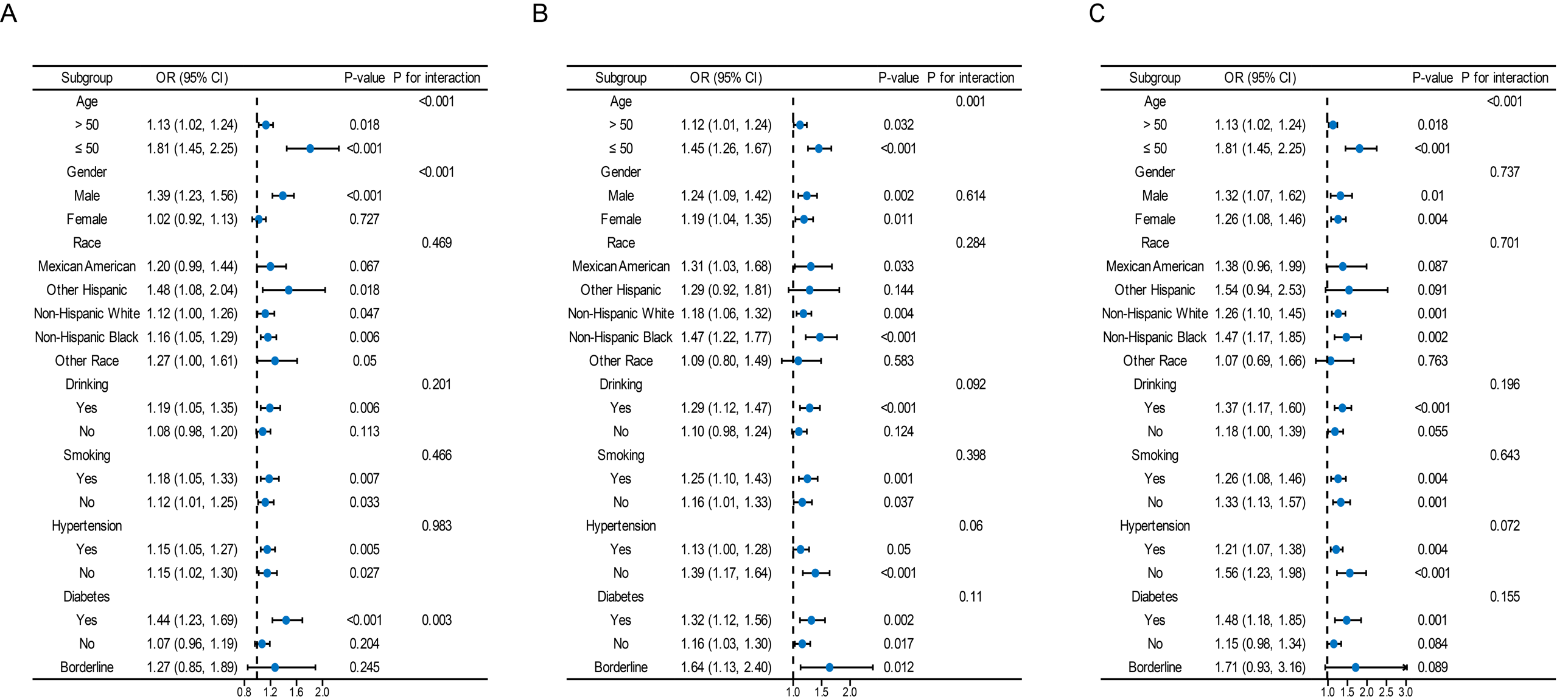


Supplementary Figure. 1. Subgroup analysis for the association between the TyG-WHtR and the risk of CKD, CVD, and CRS.

The variables adjusted in each model were the factors mentioned above except the stratification variables.

Data were listed as the weighted odd ratio estimates and 95% confidence intervals.


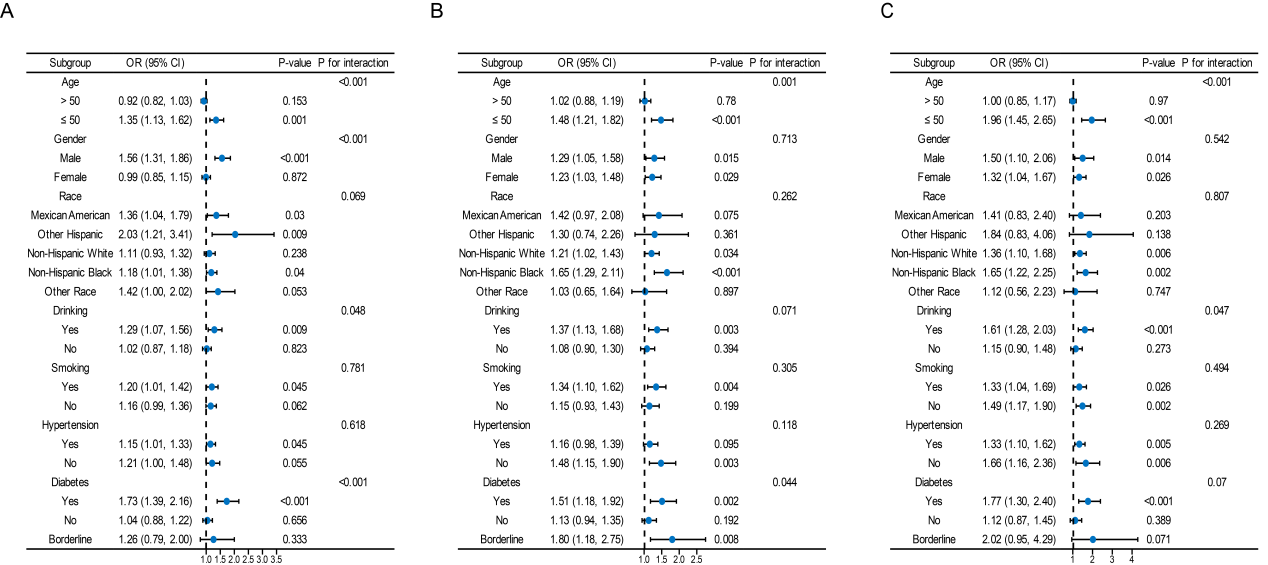


Supplementary Figure. 2. Subgroup analysis for the association between the TyG-BMI and the risk of CKD, CVD, and CRS.

The variables adjusted in each model were the factors mentioned above except the stratification variables.

Data were listed as the weighted odd ratio estimates and 95% confidence intervals.


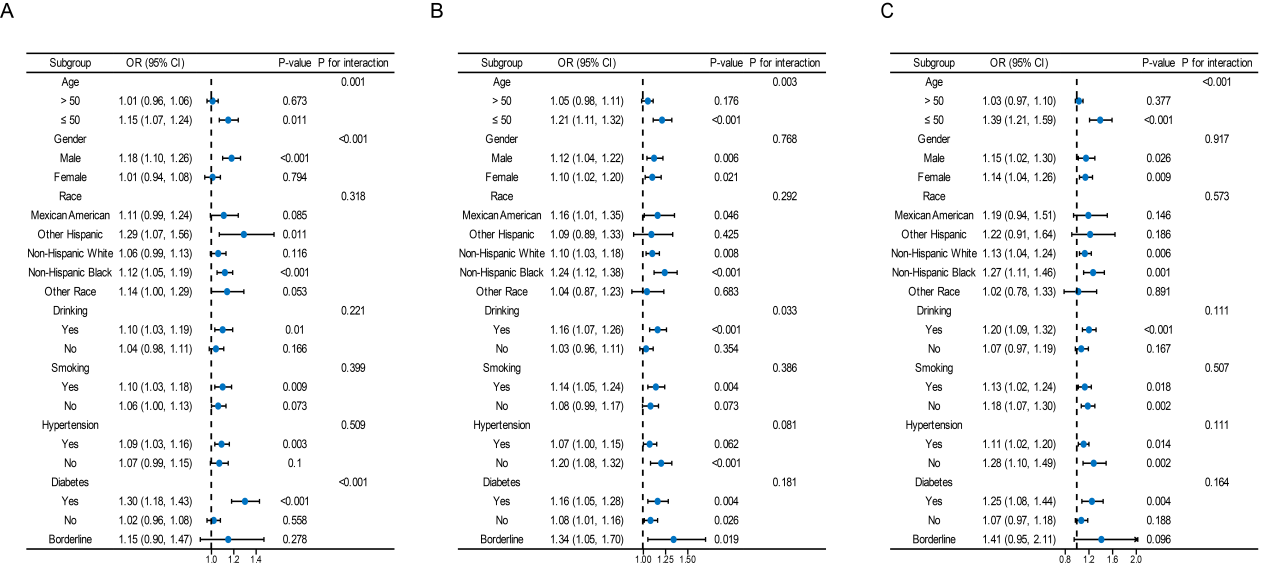


Supplementary Figure. 3. Subgroup analysis for the association between the TyG-WC and the risk of CKD, CVD, and CRS.

The variables adjusted in each model were the factors mentioned above except the stratification variables.

Data were listed as the weighted odd ratio estimates and 95% confidence intervals.
